# Supplementary material for: Mapping the evolution of fertility support policies in China: A content and instrumental analysis
Source: PLoS One. 2025 Oct 9;20(10):e0332137. doi: 10.1371/journal.pone.0332137 (PMC12510515; doi:10.1371/journal.pone.0332137)
Supplement: S1 Appendix — (ZIP) [file pone.0332137.s001.zip › S1 Appendix. 226 original policy documents/147-中国人民保险(集团)公司关于印发《中国人民保险(集团)公司机关考勤请假管理办法》的通知(FBM-CLI-6-29829).doc]

中国人民保险(集团)公司关于印发《中国人民保险(集团)公司机关考勤请假管理办法》的通知

**发布部门：** [中国人民保险公司](https://www.pkulaw.com/law/chl?Aggs.IssueDepartment=7061001)

**发文字号：**保发〔1996〕154号

**发布日期：**1996.06.26

**实施日期：**1996.07.01

**时效性：** [现行有效](https://www.pkulaw.com/law/chl?Aggs.TimelinessDic=01)

**效力级别：** [行业规定](https://www.pkulaw.com/law/chl?Aggs.EffectivenessDic=XK06)

**法规类别：** [工资福利与劳动保险](https://www.pkulaw.com/law/chl?Aggs.Category=08707)

　　　　　　 中国人民保险（集团）公司关于印发《中国人民保险
　　　　　　　　（集团）公司机关考勤请假管理办法》的通知
　　　　　 （保发（１９９６）１５４号　１９９６年６月２６日）

集团公司机关各部门：
　　现将《中国人民保险（集团）公司机关考勤请假管理办法》印发给你们，请按照文件有关规定认真做好贯彻落实工作。在执行本办法过程当中如遇有问题，可及时与集团公司人事部联系。

附：　中国人民保险（集团）公司机关考勤请假管理办法（试行）

　　为维护集团公司机关的正常办公秩序，加强劳动纪律，提高工作效率，保证集团公司各项管理和业务工作的顺利开展，根据国家的有关规定，制定本办法。
　　**一、**考勤是职工在岗和离岗的记录，是考核职工工作态度，获得劳动报酬和奖惩的重要依据之一，集团公司全体职工必须自觉遵守和严格执行考勤请假制度。

　　**二、**日常考勤的管理
　　（一）职工每日考勤以处室为单位采用到离签名方式。职工上班和下班本人应亲自在考勤簿上签字，任何他人不得代签。
　　（二）职工出差或请假，所在处室负责人须在考勤簿上签字确认。职工因故不能按时上下班的，须事先请假。
　　（三）各处室每日８∶１０前和１６∶４５后应将本处室考勤簿交综合处，由综合处确认当天的考勤记录。
　　（四）各部门应指定一名责任心强，认真负责的同志负责本部门日常考勤工作，月末将本部门考勤情况汇总，部门领导审核签字后于次月３日前送交人事部机关于部处。
　　（五）按集团公司规定的上下班时间，凡未请假或补假超过上班时间３０分钟以内到岗者，或未到下班时间提前离岗在３０分钟以内者，均按迟到或早退处理。
　　迟到或早退按月累计计算，当月累计迟到或早退合计达到５次，从第５次起，每次另按旷工１天处理。
　　（六）属于下列情况之一者，视为旷工：
　　１．未办理请假手续不到工作岗位或请假未获批准擅自离开工作岗位者；
　　２．迟到或早退时间超过３０分钟以上者；
　　３．在工作时间内，私自离开工作岗位超过３０分钟以上者；
　　４．每月累计迟到和早退合计达到５次及以上者；
　　５．假期期满后，无故不到工作岗位者；
　　６．无故不参加各类学习、会议者。
　　旷工按小时累计计算。累计旷工时间在４小时以内（含４小时）为半天，超过４小时不足８小时为１天。
　　职工连续旷工超过１５天或一年内累计超过３０天者，按国家有关规定给予辞退。
　　（七）因班车误点超过规定的上班时间到岗视为迟到或旷工。
　　（八）替他人代签的，一经发现对当事人将给予一定的处罚。

　　**三、**假期的种类及其管理
　　（一）事假及其管理
　　１．职工因私事离岗须事先请假，经有关领导批准后方能离开工作岗位，按事假对待。特殊情况本人确实不能提前办理请假手续时，可委托他人代办。假期期满后应及时销假并说明情况。
　　２．职工请假批准权按行政隶属关系由上一级领导批准。部门领导请假由公司主管总经理批准；正副处长请假由部门总经理批准；正科级及其以下职工请假在２天以内的，由本人所在处室领导批准；５天以内的，由所在部门领导批准；６天以上者，须报人事部审批。
　　３．事假按月累计计算。累计不足４小时按半天计算，累计超过４小时不足８小时按１天计算。
　　４．职工每月可享受１天带薪事假（不影响各种津补贴，影响全勤）。
　　（二）病假及其管理
　　１．职工工作时间到医院就诊，须请病假。职工因病或非因公负伤，需要治疗或休息时，应持医院医疗诊断书和休假证明办理病假手续。
　　２．病假按月累计计算。累计不足４小时的按半天计算，累计超过４小时不足８小时按１天计算。
　　３．职工病假在二个月以内的，工资照发；超过二个月的，病假期间工资待遇按下列标准发给：
　　（１）职工病假超过两个月的，从第三个月起，工作年限不满十年的，发给本人工资（等级工资与按国家规定比例计算的责任目标津贴之和，下同）的９０％；工作年限满十年的，工资照发。
　　（２）职工病假超过六个月的，从第七个月起，工作年限不满十年的，发给本人工资的７０％，工作年限满十年和十年以上的，发给本人工资的８０％。
　　４．职工在病假期间．可以继续享受公司的生活福利待遇。
　　（三）年休假及其管理
　　１．职工工作时间满一年以上的，可享受年休假。
　　２．年休假时间根据工作年限的不同分为以下几个档次：
　　（１）工作年限满一年不满五年的，每年休假５天；
　　（２）工作年限满五年不满十五年的，每年休假１０天；
　　（３）工作年限满十五年不满二十五年的，每年休假１５天；
　　（４）工作年限满二十五年以上的，每年休假２０天。
　　３．年休假期间工资照发，不影响各种津补贴。
　　４．有关具体问题的处理按照《中国人民保险（集团）公司系统职工年休假暂行办法》的规定执行。
　　（四）探亲假及其管理
　　１．职工与配偶不住在一地，又不能在公休假日团聚的，可享受探望配偶的待遇；与父母都不住在一地，又不能在公休假日团聚的，可享受探望父母的待遇。
　　２．职工休探亲假须经部门领导同意，并报人事部审批。
　　（１）未婚职工探望父母的，每年一次，假期２０天；
　　（２）已婚两地分居职工探望配偶的，每年给一方探亲假一次，假期３０天；
　　（３）已婚职工探望父母的，每四年一次，假期２０天。
　　探亲假期指职工与配偶、父、母团聚的时间，包括公休假日和法定节日在内，另外根据实际需要给予往返路程假。
　　３．职工在规定的探亲假和路程假期内，工资照发，不影响各种津补贴。
　　职工探望配偶和未婚职工探望父母的往返路费，按规定由公司负担。已婚职工探望父母的往返路费，按规定在本人工资３０％以内的由本人自理，超过部分由公司负担。
　　４．职工申请出国探亲，所在部门同意后须报人事部批准，原则上最多给予３个月假期。探望父母或配偶的，除国家另有规定者外，给予１个月的探亲假，超过１个月的时间按事假处理；探望亲友的，按事假处理。
　　（五）产假及其管理
　　１．女职工产假为９０天，多胞生育的，每多生育一个婴儿增加产假１５天。女职工怀孕不满四个月流产时，根据医务部门的意见，可给予１５天至３０天的产假；怀孕满四个月以上流产时，给予４２天的产假。
　　晚育（已婚妇女满二十四周岁后初育）的女职工，另增加奖励假３０天。对不休奖励假的，给予女方一个月的工资奖励，奖励费由夫妻双方所在单位各负担５０％。
　　对自愿生育一个子女，并领取《独生子女父母光荣证》的女职工，经批准可再增加产假９０天，但减免三年的独生子女父母奖励费。
　　２．产假期间工资照发，不影响各种津补贴。
　　３．计划生育假及其管理，按中国人民保险公司计划生育委员会关于《计划生育有关假期及奖励的规定》执行。
　　（六）婚假及其管理
　　职工持有结婚证明，经本部门领导批准，可以享受３天的婚假。女年满２３周岁、男年满２５周岁以上初婚为晚婚，双方晚婚的，另奖励晚婚假７天。
　　因对方在外地而前往结婚的，按实际需要另给往返路程假。
　　婚假期间工资照发，不影响各种津补贴。
　　（七）丧假及其管理
　　职工直系亲属（指父母、夫妻，子女以及抚养人）死亡时，经职工所在部门领导批准，给予３天丧假。在外地办丧事的，按实际需要另给往返路程假。
　　丧假期间工资照发，不影响各种津补贴。
　　（八）其他假期及管理
　　１．职工出差（含出国进修、实习）占用公休假日和法定节日的，按下列标准给予补休：
　　出差６天以上（含６天，下同）１５天以内的，给予１天补休时间；出差１６天以上３０天以内的，给予２天补休时间；出差３１天以上的，给予３天补休时间；出差半年以上，给予５天补休时间。
　　长期派驻境外机构工作（２年以上）的人员，调回国内后（在外未休假的），给予３０天休假。休假期间工资照发，不影响各种津补贴。
　　２．女职工有不满１２个月（含１２个月）婴儿的，在每天工作时间内可给哺乳假两次，每次３０分钟，每多哺乳一个婴儿，每次哺乳时间增加３０分钟。
　　家距办公地点较远的女职工，可采取每日六小时工作制。超过规定时间者，其超过部分按事假处理。
　　３．有子女入托的职工，每星期一因送子女入托最晚不能超过上班时间一小时到岗；星期五因接子女不能早于下班时间两小时离岗。
　　４．学校、幼儿园召开家长会，凭证明每次给半天公假，全年最多２次；因分房搬家，持有关证明给予２天公假。以上假期影响全勤，不影响各种津补贴。
　　５．本人申请经批准需占用工作时间学习的，每周最多不超过２个半天，影响全勤，不影响各种津补贴，超过部分按事假处理。

　　**四、**职工在探亲假、产假、婚假、丧假等期满后因病或非因公负伤不能按期到岗上班的，须持医院诊断证明书补办病假手续，按病假处理。因事不能按期到岗上班，须事前请假，经同意后按事假处理，否则按旷工对待。

　　**五、**病假、探亲假、产假和路程假，均包括公休假日和法定节日在内。事假、婚假、丧假和年休假均不含公休假日和法定节日在内。

　　**六、**请假一般应事前办理请假手续，事后要及时销假。如遇特殊情况本人无法事前亲自请假的，可委托他人代办，事后应说明情况。无正当理由未事前请假者按旷工处理。

　　**七、**为鼓励职工出全勤（无事、病假），对于半年内出全勤的职工，给予一次性奖励３００元；一年内出全勤的职工，给予一次性奖励１０００元。

　　**八、**本办法由中国人民保险（集团）公司人事部负责解释，自１９９６年７月１日起执行。原《中国人民保险公司机关职工考勒请假制度暂行规定》同时废止，凡原有关规定与本办法不一致的，均按本办法执行。

© 北大法宝：（[www.pkulaw.com](https://www.pkulaw.com/)) 专业提供法律信息、法学知识和法律软件领域各类解决方案。北大法宝为您提供丰富的参考资料，正式引用法规条文时请与标准文本核对。
欢迎查看所有[产品和服务](https://www.pkulaw.com/helps/2.html)。法宝快讯：[如何快速找到您需要的检索结果？](https://www.pkulaw.com/helps/70.html)[法宝](https://www.pkulaw.com/helps/69.html)V6有何新特色？

扫描二维码阅读原文

**原文链接：**<https://www.pkulaw.com/chl/7ed2be2ece4040c4bdfb.html>
